# Antibacterial Performance of PANI–CdS/Au Nanocomposites Compared to PANI and PANI–CdS

**DOI:** 10.3390/nano16080493

**Published:** 2026-04-21

**Authors:** Raad Al-Kilabi, Abdulameer H. Ali, Hude Al-Allaq, Elias Faraj Mohammed, Sahib Alkulaibi, Adel Alkhayatt, Hussein Al-Shabani, Thmr Ihsan, Haider Al-Hello

**Affiliations:** 1 College of Education for Women, University of Kufa, Najaf 54001, Iraq; raadh.tarsh@uokufa.edu.iq (R.A.-K.);; 2 Faculty of Dentistry, University of Cordoba, Najaf 54001, Iraq; eliasfarajmohammedden@cur.edu.iq (E.F.M.);; 3 College of Medicine, University of Alkafeel, Najaf 54001, Iraq

**Keywords:** gold nanoparticles, polyaniline, nanocomposites, UV–Vis, FTIR, XRD, antibacterial activity, CdS

## Abstract

Polyaniline-cadmium sulfide-gold (PANI-CdS-Au) nanocomposites were synthesized with varying Au loadings (0.023, 0.046, 0.092 wt%) to enhance antibacterial performance. Structural (FTIR, XRD) and morphological (FESEM) analyses confirmed successful formation, with nearly homogeneous nanoparticle distribution (27–53 nm) and slight XRD peak shifts indicating interfacial interactions between PANI, CdS, and Au. UV–Vis spectra revealed gold surface plasmon resonance and polaronic transitions consistent with PANI emeraldine base. XRD results showed the expected wurtzite CdS and fcc Au phases. Agar well diffusion tests against Escherichia coli (Gram-negative) and Staphylococcus aureus (Gram-positive) demonstrated that the 0.092 wt% of Au composite produced the largest inhibition zones at 100 µg mL^−1^ (*E. coli*: 36 mm; *S. aureus*: 24 mm), with the same trend at 25 µg mL^−1^. The results indicate that PANI–CdS/Au nanocomposites are promising antibacterial materials; however, the presence of CdS necessitates additional cytotoxicity assays to confirm their suitability for medical applications.

## 1. Introduction

Polyaniline (PANI) is a versatile conductive polymer widely used in sensors, photocatalysis, and electrochemical applications due to its excellent electrical conductivity and reducing properties [1]. While PANI–CdS nanocomposites have been studied for their optical, electrical, and photocatalytic properties, most reports focus on material characterization rather than systematic evaluation of antibacterial performance [2]. Similarly, PANI–Au nanocomposites have been explored for electronic and optoelectronic applications, but their antibacterial activity has not been comprehensively compared against CdS-based systems [3]. Previous studies on PANI-bimetal nanocomposites suggest potential antibacterial and anticancer activity, but these works did not investigate ternary systems combining PANI, CdS, and Au, nor did they quantify how Au loading influences antibacterial efficiency [4]. Numerous studies have investigated low-dimensional PANI structures and their organic–inorganic nanocomposites [4,5,6,7,8]. Among the various synthesis methods, interfacial polymerization is considered a simple and cost-effective approach for producing low-dimensional PANI [7,8].

Noble metal-based PANI nanocomposites exhibit enhanced optical and electrical properties, attributed to surface plasmon resonance and the free-electron characteristics of metallic nanostructures [9,10,11,12]. For biomedical applications, gold nanoparticles are often highlighted for their biocompatibility with human cells [13,14], ease of production [15,16], facile bioconjugation with diverse biomolecules (polyethylene glycol (PEG), carboxyl groups, amines, DNA, RNA, antibodies, peptides) [17,18], chemical stability [19], and adaptable engineering [20,21]. However, in the present system, Au is combined with CdS, which—despite its valuable optical and photocatalytic properties—contains cadmium, a potentially toxic element. This duality underscores the need for careful evaluation: while Au contributes biocompatibility and stabilization, CdS introduces possible cytotoxic risks, making biological safety assays essential for biomedical translation.

It is well established that cadmium (Cd) has no biological role and is hazardous to humans [22]. The antioxidant defense system may be impaired by reactive oxygen species (ROS) generated through oxidative stress caused by Cd bioaccumulation in human tissues, leading to various health problems [23]. Cadmium sulfide (CdS), while offering luminescent properties, superior optical/electrical characteristics, and photocatalytic activity, still contains cadmium and therefore cannot be assumed safe for biomedical applications. Although CdS exhibits lower toxicity compared to elemental Cd [24,25], its potential cytotoxicity remains a critical concern, necessitating rigorous toxicological evaluation before any biomedical use. CdS nanoparticles (NPs) can be synthesized through physical, chemical, and biological processes. Their applications depend on features such as size, shape, and surface charge, which are determined by the synthesis method [26].

Biologically synthesized CdS NPs are increasingly used in medical sciences due to their enhanced compatibility with biological systems and reduced toxicity [27]. Their non-toxicity, combined with antioxidant, antimicrobial, anticancer, imaging, and drug delivery properties, has made CdS NPs valuable as therapeutic and diagnostic tools in both in vitro and in vivo models [28,29].

In this study, a PANI–CdS/Au nanocomposite was prepared. PANI was synthesized via chemical polymerization using aniline as the monomer and ammonium persulfate (APS) as the oxidizing agent. CdS nanoparticles were subsequently synthesized using the sol–gel technique, while gold nanoparticles were produced by reduction and incorporated at varying amounts to form the ternary composite. The resulting materials were characterized using UV–Vis spectroscopy, Fourier-transform infrared spectroscopy (FTIR), X-ray diffraction (XRD), and field emission scanning electron microscopy (FESEM). The antibacterial activity of the nanocomposite was evaluated using the agar well diffusion method. The central hypothesis of this work is that incorporation of Au nanoparticles into the PANI–CdS matrix enhances antibacterial performance through plasmon-assisted generation of reactive oxygen species (ROS) and improved charge-transfer dynamics at the polymer–semiconductor–metal interface, thereby increasing bactericidal efficiency against Gram-positive and Gram-negative bacteria.

## 2. Materials and Methods

### 2.1. Synthesis of AuNPs

The Gold nanoparticles were prepared by reducing hydrated gold (III) chloride (HAuCl_4_·3H_2_O, 99%, HIMEDIA) using sodium citrate (Na_3_C_6_H_5_O_7_) as the reducing agent (Macklin Biochemical, Shanghai, China). One gram of HAuCl_4_·3H_2_O was dissolved in 200 mL of deionized water in a 500 mL volumetric flask to obtain a crude solution with a concentration of 14.7 mM. This solution was then diluted to 250 mL to achieve the desired concentration and stored in a brown bottle to prevent photodegradation.

Simultaneously, 0.5 g of Na_3_C_6_H_5_O_7_ was dissolved in 50 mL of deionized water to prepare a 1% (38 mM) sodium citrate solution. To avoid contamination, all glassware was thoroughly rinsed and cleaned with purified water prior to synthesis.

For nanoparticle preparation, 3 mL of the 1% sodium citrate solution was diluted with 250 mL of distilled water in a 500 mL Erlenmeyer flask placed on an electric stove equipped with a stirring motor. The solution was heated to 60–70 °C under continuous stirring. Subsequently, 3 mL of the 14.7 mM gold solution was added dropwise to the rapidly stirred citrate solution. The mixture gradually turned a dark crimson color, indicating the formation of gold nanoparticles. To confirm nanoparticle formation beyond visual observation, UV–Vis spectroscopy was performed immediately after synthesis, revealing the characteristic surface plasmon resonance (SPR) band at ~518 nm, which validated the successful generation of Au nanoparticles.

### 2.2. Preparation of Polyaniline (PANI) Polymer

Polyaniline (PANI) was synthesized via chemical oxidative polymerization using ammonium persulfate (APS) as the oxidizing agent and catalyst in an acidic medium. A three-neck flask was cooled to 0 °C in an ice bath, and 5 mL of aniline was dissolved in 100 mL of 1.0 M hydrochloric acid. In parallel, 50 mL of 1.0 M HCl and 15 g of APS were dissolved in a separating funnel. After 30–40 min of mixing, the two solutions were combined and stirred continuously for 24 h (Macklin Biochemical, Shanghai, China).

Upon reaching room temperature, a dark green precipitate of polyaniline was obtained. The precipitate was filtered, washed with deionized water and methanol, and dried in an oven at 353 K for 24 h, yielding polyaniline emeraldine salt (PANI-ES) as an agglomerated powder.

To prepare emeraldine base (PANI-EB), the PANI-ES was deprotonated with NH_4_OH for 15 min. Oligomers were removed by washing with methanol, and the resulting PANI-EB was dried in an oven at 60 °C for 24 h before being ground into a fine powder (Macklin Biochemical, Shanghai, China).

### 2.3. Synthesis of CdS Nanoparticles

Cadmium acetate (Cd(CH_3_COO)_2_·2H_2_O) and thiourea (CH_4_N_2_S) were used as cadmium and sulfur sources, respectively, in the sol–gel synthesis of CdS nanoparticle powder (Macklin Biochemical, Shanghai, China). A solution containing 3 g of cadmium acetate and 2 g of thiourea in 40 mL of methanol was vigorously stirred for 1 h at 60 °C, producing a gel-like substance. After heating was discontinued, the mixture was agitated until a yellow powder formed. Monocrystalline CdS nanoparticles were obtained by drying the powder at 300 °C for 30 min.

### 2.4. Synthesis of PANI–CdS/Au Nanocomposite Powder

The e appropriate amounts of PANI and CdS were dispersed in 80 mL of deionized water. To this mixture, the required volume of Au solution and acid was added, resulting in immediate reduction. The suspension was stirred for 15 min for each experiment, yielding the PANI–CdS/Au nanocomposite powder.

### 2.5. Characterization Techniques

The crystal structure was analyzed using a Philips XRD-6000 Shimadzu powder X-ray diffraction (XRD) spectrometer (Shimadzu, Kyoto, Japan). XRD patterns were recorded in the range of 10–80°, with a step width of 0.021° and a step time of 1.25 s, employing CuKα radiation (λ = 1.5406 Å). Fourier-transform infrared (FTIR) spectroscopy of PANI–CdS, and PANI–CdS/Au nanocomposites (0.023, 0.046, and 0.092 wt%) was performed in the frequency range of 400–4000 cm^−1^ using a Shimadzu FTIR-8400S (Shimadzu, Kyoto, Japan). Morphological analysis was carried out using field emission scanning electron microscopy (FESEM, Quanta 450, FEI Company, now Thermo Fisher Scientific, Brno, Czech Republic) operating at 30 kV. UV–Vis absorption and transmission spectra of PANI, PANI–CdS, and PANI–CdS/Au nanocomposites (0.023, 0.046, and 0.092 wt%) were recorded in the wavelength range of 200–1100 nm using a MEGA-2100 UV–Vis spectrophotometer (SCINCO Co., Ltd., Seoul, Republic of Korea).

### 2.6. Antibacterial Activity of PANI–CdS/Au Nanocomposites

The antimicrobial activity of PANI–CdS/Au nanocomposites (0.023, 0.046, and 0.092 wt%) was evaluated against two clinical bacterial strains: *Escherichia coli* (Gram-negative) and *Staphylococcus aureus* (Gram-positive), using the agar well diffusion (Al-Gharry Center, an-Najaf Al-Ashraf, Iraq). A standardized bacterial suspension (1.5 × 10^8^ CFU/mL, 0.5 McFarland standard) was swabbed onto Mueller–Hinton agar plates using sterile cotton swabs (BioMerieux, Craponne, France). Wells (9 mm diameter) were created using a sterilized corn borer, and 100 µg/mL and 25 µg/mL of each nanocomposite were introduced into separate wells. Plates were incubated at 37 °C for 24 h, after which inhibition zones were measured in millimeters.

## 3. Results

### 3.1. UV–Vis Analysis

**Figure 1:**
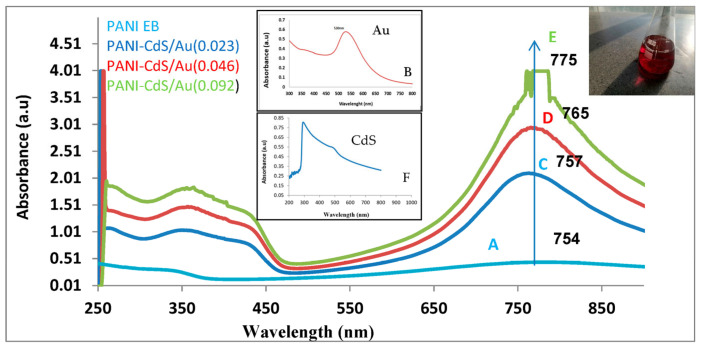
Absorbance spectra of (**A**) polyaniline (PANI) in its emeraldine base form, (**B**) gold nanoparticles (Au NPs), and (**C**–**E**) PANI-CdS/Au nanocomposites at different Au weight percentages: (**C**). 0.023 wt%, (**D**). 0.046 wt%, and (**E**). 0.092 wt%. (**F**). CdS. The spectra show characteristic absorbance peaks, with the main band observed in the range of 754–775 nm, corresponding to the nanocomposite samples. The inset (**B**) highlights the localized surface plasmon resonance (LSPR) peak of Au NPs around 518 nm.

The UV–Vis absorption spectra of gold nanoparticles and PANI–CdS/Au nanocomposites reveal distinct optical features that highlight strong electronic interactions within the hybrid system (Figure 1). The gold nanoparticles exhibit a well-defined surface plasmon resonance (SPR) band centered at ~518–530 nm, which is a characteristic signature of their formation and accounts for the ruby-red coloration of the colloidal suspension [30].

For polyaniline in its emeraldine base (EB) form, absorption bands are observed at ~343 nm and ~759 nm. The former corresponds to π–π* transitions within the conjugated backbone, while the latter is associated with polaron-related transitions in the near-infrared region. These spectral features are consistent with previous reports on PANI EB, where the lower-energy band reflects the delocalization of charge carriers and their coupling with π orbitals [29].

In the composite spectra, the incorporation of CdS and Au nanoparticles modifies the optical response significantly. The CdS absorption edge near 500 nm broadens the shoulder region in the blue–green range, while its band at ~300 nm overlaps and enhances the π–π* transition of PANI around 330 nm. Moreover, the SPR band of Au nanoparticles (~530 nm) emerges adjacent to the CdS edge, producing a synergistic spectral profile. The slight red-shift in the CdS absorption edge in the composite compared to pristine CdS suggests strong chemical coupling or charge transfer interactions between CdS domains and the polyaniline chains [31].

Upon incorporation of CdS and Au nanoparticles into the PANI matrix, the nanocomposites exhibit enhanced absorption intensity and progressive red-shifts in the near-infrared band (754–775 nm) as the Au content increases (0.023, 0.046, and 0.092 wt%). This red-shift may signify a transition toward lower-energy photon absorption, potentially arising from plasmonic–semiconductor–polymer coupling. The strengthening of the SPR band with higher Au loading is consistent with increased nanoparticle density, while the spectral evolution could hypothetically indicate improved charge-transfer dynamics between PANI, CdS, and Au [32].

At the highest Au concentration (0.092 wt%), the red-shift is most pronounced, suggesting maximized plasmonic enhancement and extended light-harvesting capability. Such behavior is particularly relevant for optoelectronic and photocatalytic applications, as the broadened absorption facilitates efficient electron transfer and may contribute to reactive oxygen species (ROS) generation, thereby potentially augmenting the nanocomposite’s antimicrobial activity [31].

### 3.2. Fourier Transform Infrared Analysis

**Figure 2:**
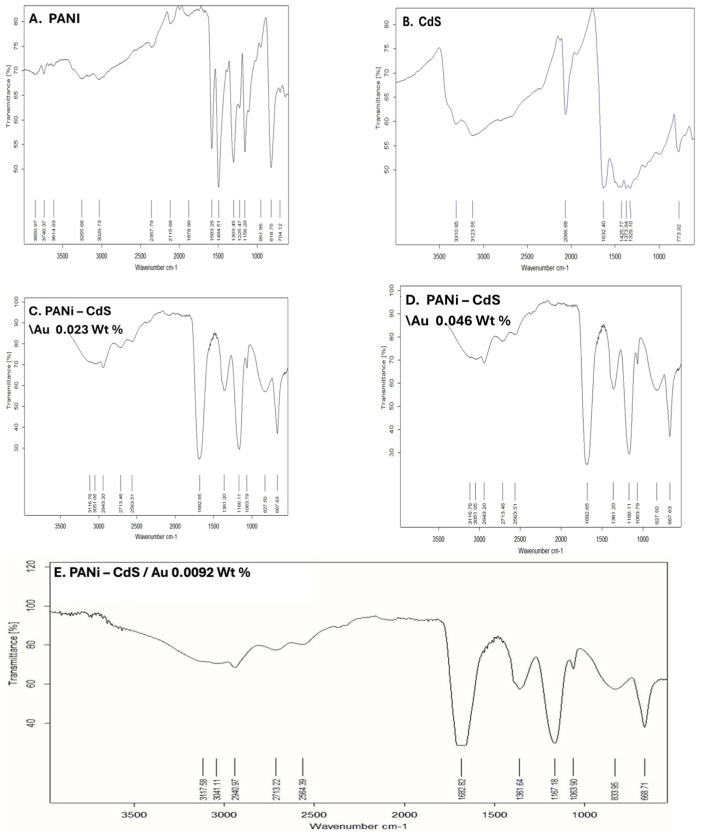
FTIR spectra of (**A**) pristine polyaniline (PANI), (**B**) cadmium sulfide (CdS) nanoparticles, and (**C**–**E**) PANI–CdS/Au nanocomposites containing Au at weight fractions of 0.023, 0.046, and 0.092, respectively.

This study presents Fourier-transform infrared (FTIR) spectra of pristine polyaniline (PANI), CdS nanoparticles, and PANI–CdS/Au nanocomposites (Figure 2). The FTIR spectrum of PANI displays the characteristic absorption bands at ~1591 and ~1491 cm^−1^, corresponding to quinoid and benzenoid ring vibrations, respectively. The band at 1381 cm^−1^ is attributed to C–N stretching of aromatic amine groups, while absorptions in the range of 1163–1061 cm^−1^ are associated with C–N^+^ stretching vibrations in the emeraldine salt structure [33]. The peak at ~827 cm^−1^, assigned to out-of-plane C–H bending, further confirms the emeraldine form of PANI.

The FTIR spectrum of CdS nanoparticles reveals a distinct absorption band at ~775 cm^−1^, which is assigned to Cd–S bond stretching. A band at ~1632 cm^−1^ corresponds to bending vibrations of adsorbed water molecules (H–O–H), while the broad absorption centered near 3310 cm^−1^ is indicative of hydroxyl (O–H) groups, most likely arising from surface-bound species [34].

In the PANI–CdS/Au nanocomposites (0.023, 0.046, and 0.092 wt% Au), gold itself does not contribute intrinsic IR-active modes. However, the spectra display organic absorptions such as a C=O stretching band near 1622–1692 cm^−1^, suggesting the presence of stabilizing organic moieties on the nanoparticle surface. The characteristic PANI bands remain conserved, though slight shifts in peak positions and intensities are observed. These spectral modifications may suggest electronic and interfacial interactions between PANI, CdS, and Au, and are consistent with the formation of a ternary nanocomposite system. The preservation of PANI’s emeraldine features alongside Cd–S and surface-bound organic absorptions confirms successful integration of CdS and Au within the polymer matrix, while the subtle spectral changes highlight synergistic interactions that may enhance charge transfer and stability.

### 3.3. Structural Analysis of PANI, CdS and PANI-CdS/Au Nanocomposites

**Figure 3:**
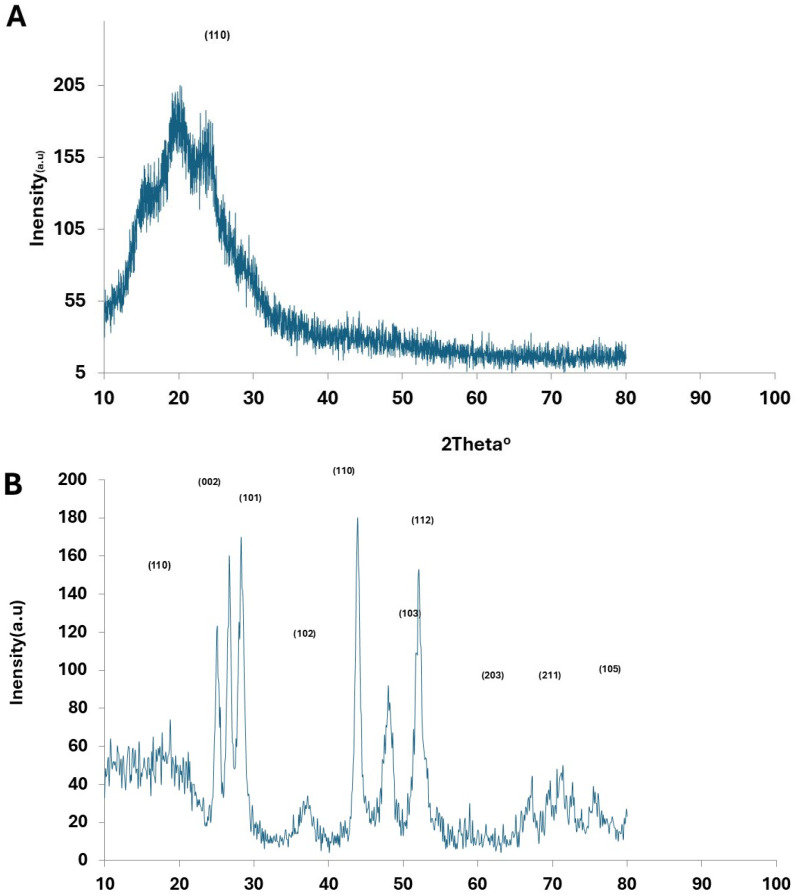

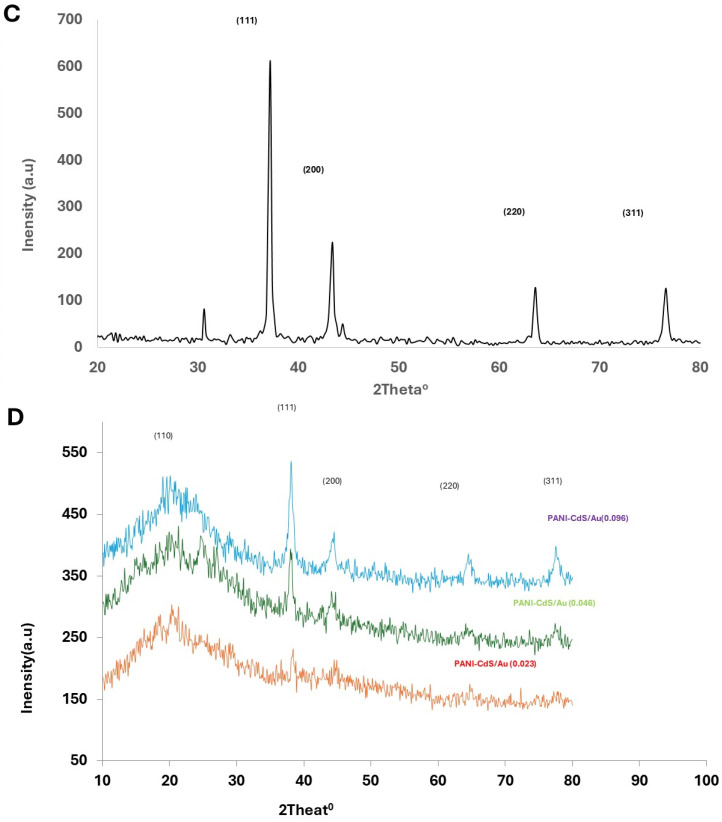
X-ray diffraction (XRD) patterns of (**A**) pristine polyaniline (PANI), (**B**) cadmium sulfide (CdS) nanoparticles, (**C**) gold nanoparticles (Au), and (**D**) PANI–CdS/Au nanocomposites containing Au at weight fractions of 0.023, 0.046, and 0.096, respectively.

The X-ray diffraction (XRD) patterns of pristine PANI, CdS nanoparticles, Au nanoparticles, and PANI–CdS/Au nanocomposites are presented (Figure 3). Pristine PANI exhibits a broad diffraction band centered at 2θ ≈ 25°, which corresponds to the periodicity parallel to the polymer chains, confirming its semi-crystalline emeraldine base structure [35].

The XRD profile of CdS nanoparticles reveals a polycrystalline hexagonal wurtzite phase, with diffraction peaks at 2θ values of ~25.0°, 26.5°, 28.0°, 43.8°, 47.8°, and 51.9°, indexed to the (100), (002), (101), (110), (103), and (112) planes, respectively (Figure 3B), in agreement with JCPDS card No. 23-0677 [36].

The diffraction pattern of Au nanoparticles displays peaks at 2θ values of 37.2°, 43.4°, 63.6°, and 76.6°, corresponding to the (111), (200), (220), and (311) planes of the face-centered cubic (fcc) structure (Figure 3C), consistent with JCPDS card No. 89-3697 [32].

**Figure 4:**
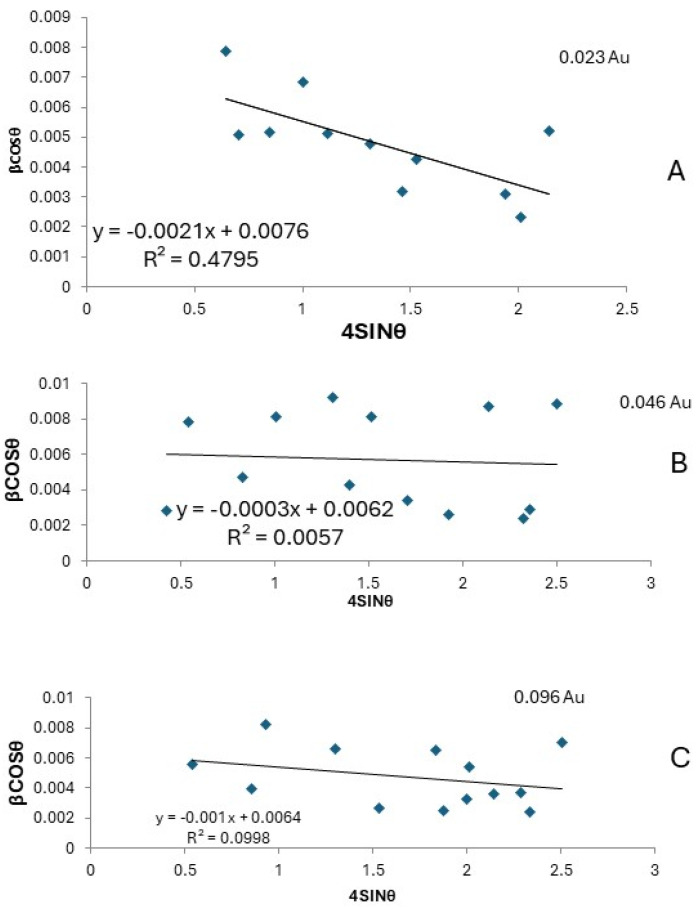
Williamson–Hall plots (βcosθ vs. 4sinθ) for PANI–CdS/Au nanocomposites at different Au concentrations: (**A**) 0.023 wt% Au: Negative slope (−0.0021) with crystallite size ~18.2 nm, indicating strong compressive strain and lattice contraction. (**B**) 0.046 wt% Au: Negative slope (−0.0003) with crystallite size ~22.4 nm, showing weaker strain but larger crystallites compared to sample A. (**C**) 0.096 wt% Au: Negative slope (−0.0010) with crystallite size ~21.7 nm, confirming persistent compressive strain and moderate lattice contraction. Although the regression coefficients (R^2^) vary, the consistent negative slopes across all samples demonstrate systematic lattice contraction due to Au incorporation and strong interfacial interactions between PANI chains and CdS/Au surfaces [38].

In the PANI–CdS/Au nanocomposites (Figure 3D), sharp and well-defined diffraction peaks are observed at 2θ ≈ 38.2°, 44.4°, and 77.3°, corresponding to the (111), (200), and (311) planes of Au nanoparticles. CdS retains its most intense peak at 2θ ≈ 28.9° (101 plane), while the reflections at ~25.9° and ~26.7° overlap with the broad PANI band, resulting in a shoulder-like feature. The embedded Au nanoparticles preserve their cubic structure; however, systematic rightward shifts in the (111) diffraction peak are observed across all composites. To quantify this effect, Williamson–Hall analysis was performed at different Au loadings (0.023, 0.046, and 0.096 wt%). The W–H plots (βcosθ vs. 4sinθ) yielded negative slopes (−0.0021, −0.0003, and −0.001, respectively), consistent with compressive microstrain and lattice contraction at the PANI–CdS/Au interfaces (Table 1 and Figure 4). The extracted crystallite sizes ranged from 18.2 to 22.4 nm, in agreement with Scherrer estimates. Although the goodness of fit varied (R^2^ = 0.48, 0.005, 0.099), the consistent negative slopes across all samples confirm that the contraction is systematic and attributable to strong interfacial interactions between PANI chains and CdS/Au surfaces.

At higher Au loading (0.096 wt%), the diffraction peaks become sharper and more intense, suggesting reduced lattice defects and enhanced crystallinity. The lattice constant for Au nanoparticles in the (111) plane was calculated as a = b = c = 4.185 Å. In the PANI–CdS/Au nanocomposites, the lattice constant decreased to 4.060 Å at 0.023 wt% and slightly increased to 4.096 Å at 0.096 wt%, reflecting subtle strain relaxation.

Crystallite sizes were estimated using the Scherrer equation. For Au nanoparticles, the average crystallite size in the (111) plane was ~21.6 nm. In the PANI–CdS/Au nanocomposites, crystallite sizes decreased progressively with increasing Au content: 29 nm (0.023 wt%), 17.4 nm (0.046 wt%), and 15 nm (0.096 wt%). The corresponding dislocation densities, calculated using Equation (2), increased with higher Au loading (1.19 × 10^15^, 3.31 × 10^15^, and 4.44 × 10^15^ cm^−2^), confirming the inverse relationship between crystallite size and dislocation density [37]. Microstrain, determined using Equation (3), was 0.0016 for pure Au nanoparticles, with lower values observed in the nanocomposites (Table 2). However, no statistically significant correlation was observed between crystallite size and antibacterial activity, which can be attributed to the narrow size distribution (18–22 nm) and particle agglomeration effects. Moreover, the low R^2^ values and negative slopes in the Williamson–Hall plots (Table 1 and Figure 4A–C) indicate irregular compressive strain, suggesting that antibacterial performance is more closely linked to surface energy and lattice defects than to crystallite size alone.

The observed peak broadening in the ternary composites is attributed not only to nanoscale effects but also to microstrain arising from mismatches in thermal expansion and lattice parameters among the metallic, semiconducting, and polymeric phases. These findings confirm successful integration of CdS and Au within the PANI matrix, with interfacial interactions enhancing crystallinity and potentially improving charge transport properties.

(1) $$D_{\mathrm{av}}=\frac{k\lambda }{\beta cos\theta }$$

(2) $$\delta =\frac{1}{{D_{av}}^{2}}$$

(3) $$\mathrm{Micro}\text{-}\mathrm{Strain}=\frac{\left(d_{XRD}-d_{JCPDS}\right)}{d_{JCPDS}}$$

(4) $$d_{hkl=\frac{a}{\sqrt{h^{2}+k^{2}+l^{2}\;}}}$$

(5) $$\frac{1}{d_{hkl}^{2}}=\frac{4}{3}\left(\frac{h^{2}+hk+k^{2}}{a^{2}}\right)+\frac{l^{2}}{c^{2}}$$

### 3.4. Morphological Analysis

**Figure 5:**
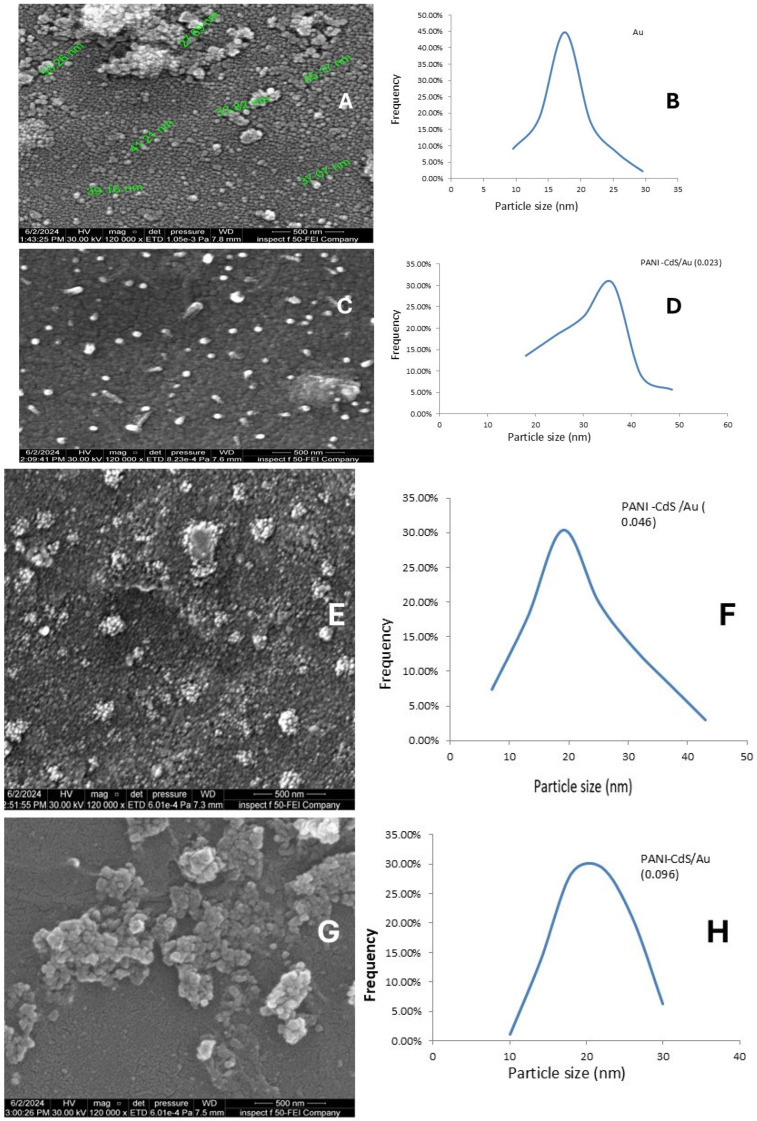
FESEM micrographs and particle size distribution histograms of Au nanoparticles and PANI–CdS/Au nanocomposites at different Au loadings. (**A**,**B**) Pure Au nanoparticles showing spherical morphology and a narrow size distribution centered at ~17 nm. (**C**,**D**) PANI–CdS/Au (0.023 wt%) composite with sparsely dispersed Au nanoparticles partially embedded in the matrix, distribution peak ~30 nm. (**E**,**F**) PANI–CdS/Au (0.046 wt%) composite with denser nanoparticle dispersion and enhanced interfacial adhesion, distribution peak ~20 nm. (**G**,**H**) PANI–CdS/Au (0.096 wt%) composite with abundant, uniformly dispersed nanoparticles and minimal agglomeration, distribution peak ~25 nm.

Field emission scanning electron microscopy (FESEM) images provide detailed insight into the morphology of Au nanoparticles and their incorporation within the PANI–CdS matrix. Pure Au nanoparticles (Figure 5A) exhibit a uniform spherical morphology, with individual particle diameters ranging between ~27 and ~64 nm as observed by SEM. To provide a quantitative assessment, particle size distribution analysis was performed (Figure 5B), revealing a narrow Gaussian distribution centered at ~17.5 nm. This distribution is consistent with the ruby-red color characteristic of colloidal Au nanoparticles in this size regime. The presence of small, well-dispersed particles with limited agglomeration confirms their colloidal stability and suitability for integration into hybrid nanostructures. Moreover, higher Au loadings (≥0.046 wt%) yielded smaller average particle sizes (~19–20 nm) and narrower distributions, reflecting improved monodispersity and stabilization by PANI chains. These findings highlight the critical role of Au concentration in controlling particle size and distribution, with implications for optimizing antibacterial performance through enhanced surface area and defect-mediated interactions.

In the PANI–CdS/Au nanocomposites (Figure 5A–H), SEM micrographs and particle size distribution histograms reveal the progressive influence of Au loading on nanoparticle morphology and dispersion. Pure Au nanoparticles (Figure 5A,B) exhibit a spherical shape with a narrow size distribution centered at ~17 nm, confirming successful synthesis and colloidal stability. Upon incorporation into the PANI–CdS framework at low Au content (0.023 wt%, Figure 5C,D), the nanoparticles appear sparsely dispersed and partially embedded within the polymer–semiconductor matrix, suggesting initial nucleation and stabilization by PANI chains. At intermediate loading (0.046 wt%, Figure 5E,F), the distribution becomes denser, with nanoparticles forming intimate contact with CdS domains, indicative of enhanced interfacial adhesion and reduced agglomeration. At higher loading (0.096 wt%, Figure 5G,H), the nanoparticles are more abundant and uniformly dispersed, with a narrow distribution centered at ~25 nm, reflecting effective stabilization by the PANI matrix and improved monodispersity.

This systematic improvement in nanoparticle dispersion and interfacial integration is critical for functional performance. The high surface-to-volume ratio maintained across all loadings maximizes effective contact between the nanocomposite and external environments, which is particularly relevant for antimicrobial activity. The morphological evidence corroborates the XRD and FTIR findings, confirming successful embedding of Au nanoparticles into the PANI–CdS framework and highlighting synergistic structural interactions that enhance charge transfer, stability, and bioactivity [39].

### 3.5. Antibacterial Activity of PANi-CdS/Au Nanocomposites

**Figure 6:**
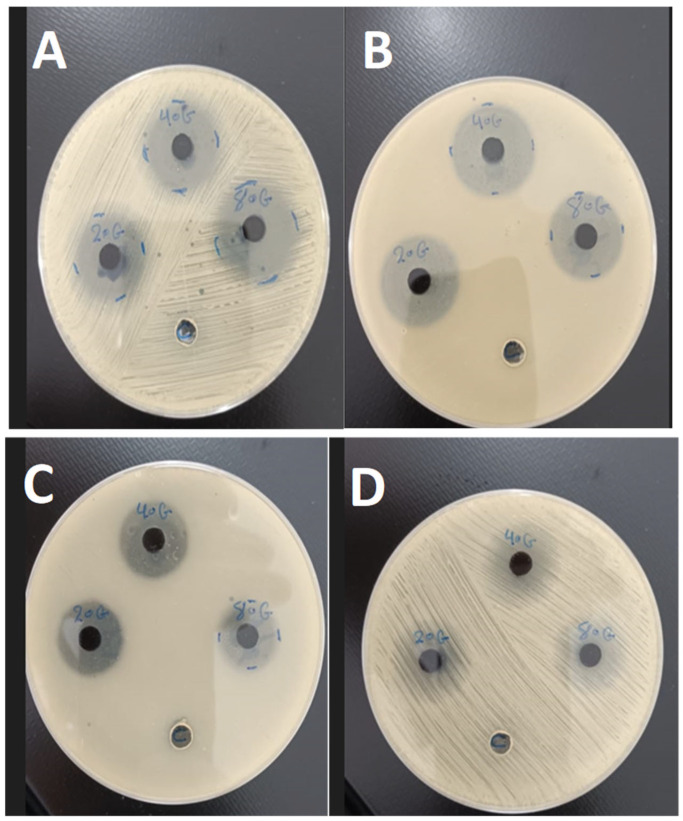
Antibacterial activity of PANI–CdS/Au nanocomposites containing Au at weight fractions of 0.023, 0.046, and 0.092, evaluated by the agar diffusion method. Inhibition zones were measured at a concentration of 100 μg/mL against (**A**) *Staphylococcus aureus* and (**B**) *Escherichia coli*, and at 25 μg/mL against (**C**) *E. coli* and (**D**) *S. aureus*.

The antibacterial performance of PANI–CdS/Au nanocomposites with varying Au nanoparticle loadings (0.023, 0.046, and 0.092 wt%) was assessed using the agar well diffusion method against *Staphylococcus aureus* (Gram-positive) and *Escherichia coli* (Gram-negative) (Figure 6). Clear inhibition zones were observed around the wells, confirming effective bactericidal activity. The nanocomposites demonstrated stronger inhibitory effects against *E. coli*, which can be attributed to its thinner and more permeable cell wall compared to the thicker peptidoglycan layer of *S. aureus*. Agar diffusion was selected as a preliminary assay due to the colloidal and non-soluble nature of the PANI–CdS/Au composites, which cause optical interference in turbidity-based MIC/MBC measurements and complicate accurate endpoint determination. Moreover, the agar matrix provides more uniform nanoparticle distribution compared to liquid media, where agglomeration can occur. These considerations justify the use of agar diffusion in this stage of the study, while MIC and MBC assays will be pursued in future work to provide quantitative validation.

The enhanced antibacterial activity is consistent with the synergistic electronic interactions among PANI, CdS, and Au, as indicated by UV–Vis redshift analysis. Smaller crystallite sizes provide a higher surface-to-volume ratio, increasing the density of active sites available for interaction with bacterial membranes. According to previous reports, such sites are known to facilitate the generation of reactive oxygen species (ROS), leading to oxidative stress and subsequent cell death. This size-dependent inhibitory trend is in agreement with earlier findings by, who demonstrated that antibacterial efficacy is inversely proportional to crystallite size. While these observations suggest that ROS generation may contribute to the antibacterial activity of PANI–CdS/Au nanocomposites, direct ROS detection was not performed in this study, and further work is required to confirm the mechanism [40,41].

The antibacterial mechanism involves direct nanoparticle–cell wall interactions, leading to encapsulation, rupture, and cytoplasmic leakage. Nanoparticles adhere to bacterial surfaces, impairing permeability and disrupting membrane integrity. In addition, polyaniline contributes intrinsic antibacterial properties through multiple mechanisms: surface hydrophilicity [42], extended polymer chain length [35], low molar mass [43], electrostatic attraction between positively charged PANI and negatively charged bacterial surfaces [44], and the presence of amino groups that enhance binding interactions [45,46].

Overall, the FESEM, XRD, and FTIR findings corroborate the antimicrobial assay results, confirming that the structural integration of Au nanoparticles within the PANI–CdS matrix enhances stability, charge transfer, and bactericidal efficiency. The observed increase in inhibition zone diameters with higher Au loading highlights the potential of these nanocomposites for biomedical and antimicrobial applications (see Table 3).

## 4. Discussion

The structural, spectroscopic, and morphological analyses collectively confirm the successful integration of CdS and Au nanoparticles within the PANI matrix. XRD results revealed distinct diffraction peaks corresponding to Au and CdS phases, with noticeable shifts in the (111) plane of Au, indicating lattice contraction and strong interfacial interactions between the metallic nanoparticles and the polymer backbone [27,28,36]. Similar lattice strain effects have been reported in other polymer–metal nanocomposites, where interfacial coupling modifies crystallinity and enhances charge transfer [32,47]. FTIR spectra further supported this observation, showing conserved PANI vibrational bands with slight intensity variations, consistent with electronic coupling among PANI, CdS, and Au [34,35].

UV–Vis absorption spectra demonstrated a pronounced redshift at higher Au loading (0.092 wt%), suggesting enhanced electron delocalization and improved light-harvesting capability. Comparable redshifts have been observed in Au–polymer hybrids [48,49], where plasmonic interactions extend absorption into the visible range and facilitate efficient electron excitation. The FE-SEM micrographs confirmed a uniform distribution of spherical Au nanoparticles within the PANI–CdS matrix, with minimal agglomeration even at the highest loading. This morphological stability is consistent with reports that conductive polymers act as effective stabilizers, preventing nanoparticle aggregation [49].

The antimicrobial assays revealed superior activity against *Escherichia coli* compared to *Staphylococcus aureus*, attributable to differences in cell wall structure [40,41]. This trend aligns with previous studies on PANI–CdS nanocomposites [32], which also reported stronger inhibition against Gram-negative bacteria. The synergy between PANI, CdS, and Au enhances the generation of reactive oxygen species (ROS), disrupting bacterial membranes and cytoplasmic integrity [9,10,19]. Gold nanoparticles act as catalytic centers, facilitating charge transfer and suppressing electron–hole recombination, thereby amplifying ROS production [11,32]. Polyaniline contributes additional antibacterial functionality through its hydrophilic surface, electrostatic interactions, and amino group chemistry [35,42,43,44,45,46].

At 0.092 wt% Au, the nanocomposite exhibited the highest antimicrobial efficiency, underscoring the importance of optimized nanoparticle loading. However, limitations must be acknowledged. First, the antibacterial assays relied on agar diffusion, which is qualitative and diffusion-dependent; quantitative MIC/MBC assays are required to validate these findings. Second, the particle size range was relatively narrow, which restricts statistical correlation between crystallite size and antibacterial activity. Finally, while the present study demonstrates promising synergy, long-term stability and cytotoxicity assessments are necessary before biomedical translation. Despite these limitations, the combination of structural stability, efficient charge transfer, and enhanced ROS generation positions PANI–CdS/Au nanocomposites as strong candidates for applications in water sterilization, antibacterial coatings, and biomedical devices. This work contributes to the growing body of literature on polymer–semiconductor–metal hybrids, highlighting how synergistic integration can advance next-generation antibacterial formulations.

## 5. Conclusions

The present study confirms the successful integration of CdS and Au nanoparticles within the PANI matrix, as evidenced by XRD lattice shifts, FTIR band preservation with slight intensity variations, and UV–Vis redshifts at higher Au loading. Morphological analysis demonstrated uniform dispersion of Au nanoparticles with minimal agglomeration, maintaining a high surface-to-volume ratio. Antibacterial assays revealed enhanced inhibition against *E. coli* compared to *S. aureus*, consistent with differences in cell wall structure. The improved activity at 0.092 wt% Au highlights the role of optimized nanoparticle loading in strengthening interfacial interactions and reactive oxygen species generation.

Within the limits of the present work, these findings establish the structural stability and antibacterial potential of PANI–CdS/Au nanocomposites. Further studies, including MIC/MBC assays, cytotoxicity evaluation, and long-term stability testing, are required before biomedical or environmental applications can be fully validated.

### Limitations and Future Work

The antibacterial assays in this study were limited to agar diffusion, which is qualitative and diffusion-dependent; quantitative MIC/MBC assays are required to validate these findings. The narrow crystallite size range restricted statistical correlation with antibacterial activity, and cytotoxicity as well as long-term stability assessments remain necessary before biomedical translation. Future work will therefore focus on (i) performing MIC/MBC assays with optimized protocols, (ii) evaluating cytotoxicity in mammalian cell models, and (iii) assessing stability and performance under real environmental and biomedical conditions.

Within these boundaries, the present findings establish the structural stability and antibacterial potential of PANI–CdS/Au nanocomposites, providing a foundation for further investigation into their suitability for advanced biomedical and environmental applications.

## Figures and Tables

**Table 1:** Structural parameters of PANI–CdS/Au nanocomposites at different Au concentrations (0.023, 0.046, and 0.096 wt%), obtained from XRD data using Scherrer and Williamson–Hall methods.

Sample (Au wt%)	Crystallite Size D (nm)	Type of Reaction (Slope)	Data Accuracy (R^2^)	Qualitative Description
0.023	18.24	Strong contraction (−0.0021)	0.479	Best fit
0.046	22.36	Slight contraction (−0.0003)	0.005	Very weak fit
0.096	21.66	Moderate contraction (−0.0010)	0.099	Weak fit

**Table 2:** The Crystallite Size, Lattice constant, Dislocation Density, Fine Strain, Au NPS and PANI-CdS/Au Nanocomposites for (111) Plane.

Material	Crystallite Size (nm)	a = b = c (A^0^)	δ line/m^2^ × 10^15^	Staring
Au NPS	21.6	4.185	2.143	0.0016046
PANI–CdS\Au (0.023)	29	4.060	1.189	0.002305
PANI–CdS\Au (0.046)	17.39	4.087	3.306	0.0047803
PANI–CdS\Au (0.092)	15	4.096	4.444	0.007970

**Table 3:** The Crystallite Size, Lattice constant, Dislocation Density, Fine Strain, Au NPS and PANI-CdS/Au Nanocomposites for (111) Plane.

Bacteria	Concentration of (100 $\frac{\mu g}{mL}$) (PANI/CdS-Au)Wt% Inhibition Zone (mm)		
	PANI/CdS-Au(0.023)Wt%	PANI/CdS-Au(0.046)Wt%	PANI/CdS-Au(0.092)Wt%
*Staph. ureus*	20	22	24
*E. coli*	23	27	36
*Staph. ureus*	15	16	18
*E. coli*	17	18	20

## Data Availability

The original contributions presented in this study are included in the article. Further inquiries can be directed to the corresponding author.
